# Retinoid Signaling in Pancreatic Cancer, Injury and Regeneration

**DOI:** 10.1371/journal.pone.0029075

**Published:** 2011-12-29

**Authors:** Emily K. Colvin, Johana M. Susanto, James G. Kench, Vivienna N. Ong, Amanda Mawson, Mark Pinese, David K. Chang, Ilse Rooman, Sandra A. O'Toole, Davendra Segara, Elizabeth A. Musgrove, Robert L. Sutherland, Minoti V. Apte, Christopher J. Scarlett, Andrew V. Biankin

**Affiliations:** 1 Cancer Research Program, Garvan Institute of Medical Research, Darlinghurst, Sydney, Australia; 2 Department of Tissue Pathology and Diagnostic Oncology, Royal Prince Alfred Hospital, Central Clinical School, University of Sydney, Camperdown, Australia; 3 Division of Surgery, Bankstown Hospital, Eldridge Road, Bankstown, Sydney, Australia; 4 St Vincent's Clinical School, Faculty of Medicine, The University of New South Wales, Australia; 5 Pancreatic Research Group, South Western Sydney Clinical School, School of Medical Sciences, The University of New South Wales, Australia; 6 School of Environmental and Life Sciences, University of Newcastle, Ourimbah, Australia; Northwestern University, United States of America

## Abstract

**Background:**

Activation of embryonic signaling pathways quiescent in the adult pancreas is a feature of pancreatic cancer (PC). These discoveries have led to the development of novel inhibitors of pathways such as Notch and Hedgehog signaling that are currently in early phase clinical trials in the treatment of several cancer types. Retinoid signaling is also essential for pancreatic development, and retinoid therapy is used successfully in other malignancies such as leukemia, but little is known concerning retinoid signaling in PC.

**Methodology/Principal Findings:**

We investigated the role of retinoid signaling *in vitro* and *in vivo* in normal pancreas, pancreatic injury, regeneration and cancer. Retinoid signaling is active in occasional cells in the adult pancreas but is markedly augmented throughout the parenchyma during injury and regeneration. Both chemically induced and genetically engineered mouse models of PC exhibit a lack of retinoid signaling activity compared to normal pancreas. As a consequence, we investigated Cellular Retinoid Binding Protein 1 (CRBP1), a key regulator of retinoid signaling known to play a role in breast cancer development, as a potential therapeutic target. Loss, or significant downregulation of CRBP1 was present in 70% of human PC, and was evident in the very earliest precursor lesions (PanIN-1A). However, *in vitro* gain and loss of function studies and CRBP1 knockout mice suggested that loss of CRBP1 expression alone was not sufficient to induce carcinogenesis or to alter PC sensitivity to retinoid based therapies.

**Conclusions/Significance:**

In conclusion, retinoid signalling appears to play a role in pancreatic regeneration and carcinogenesis, but unlike breast cancer, it is not mediated directly by CRBP1.

## Introduction

Increasing evidence supports a pivotal role for developmental signaling pathways such as Notch [1] and Hedgehog [2], [3], [4] in pancreatic cancer, with inhibitors of these pathways currently in early phase clinical trials. Notch and hedgehog are also involved in pancreatic injury, regeneration and repair [5], conditions that are known to predispose to cancer. Retinoid signaling is vital for embryonic pancreas formation [6], [7], [8], but little is known about its potential role in pancreatic cancer.

Retinoids are naturally occurring or synthetic Vitamin A analogues, which have been used successfully in the treatment of acute promyelocytic leukaemia (APL) [9]. Despite the success of retinoid treatment in APL, treatment of solid tumors has met with limited success [10], [11], and some evidence suggests that this resistance to retinoid therapy may be due to aberrations in retinoid signaling. Downregulation of retinoid receptors has been reported in many cancers, such as breast, lung, prostate and esophageal cancer [11], and downregulation of upstream components involved in retinoid metabolism and storage are emerging as possible key regulators of carcinogenesis and contributors to resistance to retinoid based therapies.

Cellular Retinoid Binding protein 1 (CRBP1) plays a major role in retinoid signaling and downregulation of CRBP1 expression occurs in breast [12], prostate [13], gastric [14] and ovarian [15] cancers. Loss of CRBP1 expression in breast epithelium leads to loss of differentiation and tumour progression by interfering with retinoid storage and its metabolism to the active metabolite, Retinoic Acid, producing a localised retinoid deficiency [16], with altered retinoid responsiveness [17] and cellular transformation.

Pancreatic cancer (PC) is the fourth leading cause of cancer death in Western societies with an overall 5-year survival rate of less than 5% [18]. Advances in neoadjuvant and adjuvant chemotherapeutic regimens have resulted in some improvement in outcomes, but pancreatectomy remains the single most effective treatment modality for PC, and offers the only potential for cure. Only 20% of patients present with localised, non-metastatic disease which is suitable for resection [19]. Those who undergo resection and receive adjuvant therapy have a median survival of 12–22 months and a 5-year survival of 20–25% [20]. Existing systemic therapies are only modestly effective and the median survival for patients with metastatic disease remains 6 months. Consequently there is a great need to develop novel therapeutic strategies for pancreatic cancer.

Here we identify a potential role for retinoid signaling in pancreatic cancer and pancreatic regeneration and as a consequence may constitute a targetable mechanism for the development of novel therapeutic strategies. Loss of CRBP1, a key regulator of retinoid signalling and important in breast cancer, although frequent in PC, unlike breast cancer was not itself sufficient to induce transformation.

## Methods

### Ethics Statement

Ethical approval for animal experimentation was obtained from the Garvan Institute Animal Ethics Committee (Approval numbers 09/53; 07/10). Multicentre ethical approval was obtained from the Human Research Ethics Committees from University teaching hospitals: Westmead Hospital, Concord Hospital, Royal Prince Alfred Hospital and St Vincent's Hospital Campus in Sydney for the acquisition of fresh and archival tissue and recording of clinicopathological data for patients with the diagnosis of pancreatic cancer. Informed written consent was obtained from all patients.

### RARE-LacZ mice

RARE-LacZ mice contain a LacZ transgene controlled by a retinoic acid response element (RARE). Cells with active RA signalling stain positive for β-galactosidase (β-gal). Untreated animals (n = 8) were sacrificed at 10–12 weeks of age and their pancreas harvested for β-gal staining.

### Murine Pancreatitis Model

A caerulein-induced model of chronic pancreatitis was used similar to previous models [21]. Mice (n = 16) were treated with repeated intra-peritoneal injections of caerulein (MP Biomedicals, Solon, OH) five times a day, twice a week for 10 weeks. In this model complete acinar cell regeneration is reported to occur by 6 weeks after cessation of caerulein treatment. Therefore mice were sacrificed at 0, 2, 4 and 6 weeks after the cessation of caerulein injections and their pancreas harvested in order to investigate RA signalling at various time points during the recovery period.

### Murine Pancreatic Cancer Models

RARE-LacZ mice (n = 10) 10 weeks of age were treated with 7,12-dimethylbenzanthracene (DMBA; Sigma-Aldrich, St Louis, MO) as previously described [22]. Mice were sacrificed when they demonstrated signs of illness or 9 months post-DMBA treatment and tissue collected for β-gal staining.

LSL-Kras^G12D/+^, LSL-Trp^53R172H/+^, and Pdx1-Cre mice were obtained from the Mouse Models of Human Cancer Consortium at the National Cancer Institute (Frederick, MD). To generate pancreatic tumours in which RA signalling activity could be assessed RARE-LacZ mice were crossed with LSL-Kras^G12D/+^;LSL-Trp^53R172H/+^;Pdx1-Cre mice to generate LSL-Kras^G12D/+^;LSL-Trp^53R172H/+^;Pdx1-Cre;RARE-LacZ offspring (n = 8). Quadruple transgenic mice were left to age until tumours formed at which point they were euthanised and tissue was collected for β-gal staining.

### β-galactosidase staining

4 µm pancreatic sections were de-waxed and rehydrated before unmasking was achieved using target-retrieval solution (S2367, pH 9.0: DAKO Corporation, Carpenteria, CA) in a boiling water bath for 20 minutes. Endogenous peroxidase activity was quenched with 3% hydrogen peroxide in methanol. Sections were incubated for 1 hour with 1∶50 anti-β-galactosidase (AB616) polyclonal rabbit antibody (Abcam, Cambridge, UK). A labelled polymer horseradish peroxidase anti-rabbit detection system was used according to the manufacturer's instructions (Envision+ anti-rabbit; DAKO Corporation, CA). 3,3′-diaminobenzidine was used as a substrate. Counter-staining was performed with Mayer's hematoxylin (DAKO Corporation, Carpenteria, CA).

### Patient Cohort

We identified a cohort of 90 patients who underwent pancreatic resection or biopsy from these hospitals. This cohort represents a subset of a previously described group of 348 patients [23], [24].

### CRBP1 Immunohistochemistry

Pancreatic tissue micro-arrays (4 µm sections) were de-waxed and rehydrated before unmasking was achieved using target-retrieval solution (S1699, pH 6.0: DAKO Corporation, Carpenteria, CA) in a pressure cooker for 5 minutes. Endogenous peroxidase activity was quenched with 3% hydrogen peroxide in methanol. Sections were incubated for 1 hour with 1∶50 anti-CRBP1 (FL-135) polyclonal rabbit antibody (Santa Cruz Biotechnology, Santa Cruz, CA). A labelled polymer horseradish peroxidase anti-rabbit detection system was used according to the manufacturer's instructions (Envision+ anti-rabbit; DAKO Corporation, Carpenteria, CA). 3,3′-diaminobenzidine was used as a substrate. Counter-staining was performed with Mayer's hematoxylin (DAKO Corporation, Carpenteria, CA).

Up to three separate samples of pancreas were examined per patient. Staining was assessed by two separate observers for each case (E.K.C. and S.A.O.). Standardisation of scoring was achieved by comparison of scores between observers, and by conferencing, where any discrepancies were resolved by consensus. Scores were given as a percentage of cells with positive cytoplasmic staining within the representative area of the tissue micro-array core, and the absolute intensity of cytoplasmic staining on a scale of 0 to 3 (0 representing no staining, 1 representing heterogenous cytoplasmic staining, 2 representing homogenous cytoplasmic staining, and 3 representing intense homogenous cytoplasmic staining). A positive score for CRBP1 was given based on the following criteria: >50% of cells having homogeneous cytoplasmic staining, with an intensity of >1.

Survival analysis was performed using Kaplan-Meier analysis, with differences in survival assessed using the Log-Rank test using StatView 5.0 Software (Abacus Systems, Berkeley, CA).

### Pancreatic Cell Lines

Six pancreatic cancer cell lines were used: AsPC-1, BxPC-3, Capan-2, HPAC, MiaPaCa2 and PANC1 (ATCC, VA, USA). These cell lines were cultured according to ATCC protocols. Human Pancreatic Ductal Epithelial (HPDE and HPDE-EcoR) cells were used as a normal control (kindly provided by Ming-Sound-Tsao, Ontario Cancer Institute) and cultured as described previously [25].

### Western blotting

Protein extracts were visualised using 4–12% Bis-Tris precast gels (Invitrogen, Victoria, Australia) and transferred to PVDF membranes according to the manufacturer's protocol. The filters were blocked with 5% skim milk in TBST buffer. Anti-CRBP1 antibody (FL-135) was used at 1∶500 overnight. Membranes were then incubated with anti-rabbit immunoglobulin G (IgG) conjugated with horseradish peroxidase (1∶2000) (Amersham, (GE Healthcare), NSW, Australia) for 1 hour at room temperature followed by enhanced chemiluminescence reagent (Perkin Elmer, Waltham, MA).

### RNA extraction and cDNA synthesis

RNA from pancreatic cell lines was extracted using Trizol® Reagent (Invitrogen, Victoria, Australia) according to the manufacturer's instructions. cDNA was synthesised using ABgene® Reverse-IT™ RTase Blend (ABgene, Surrey, UK) with a mix of Oligo dT and Random Hexamers using the First Strand Synthesis Method. Following denaturation of the RNA at 70°C, Reverse Transcription (RT) reactions were performed in a thermocycler DNA engine DYAD™ (MJ Research, Waltham, MA) with the stepwise program of 25°C for 10 minutes, 35°C for 30 minutes, 47°C for 45 minutes, then 75°C for 10 minutes.

### Quantitative Real-Time PCR (QPCR)

TaqMan® gene expression assays (Applied Biosystems, Victoria, Australia) of CRBP1 (Hs00161252_m1) were performed according to the manufacturer's instructions. GAPDH (4326317E, TaqMan® endogenous control) was used as an endogenous control. TaqMan® qPCR reactions were performed in triplicate using the ABI Prism 7900HT Sequence Detection System (Applied Biosystems, Victoria, Australia). Standard curves were included to ensure the reactions were performed in the linear range and were used to adjust the efficiency of the reaction. The cycling conditions included initial denaturation step at 95°C for 4 minutes, followed by 95°C (30 seconds) and 72°C (30 seconds) for 60 cycles.

### Knockdown of CRBP1 in HPDE cells

Two short hairpin RNA (shRNA) constructs targeting CRBP1, siCRBP1-A and siCRBP1-B as well as a scrambled control, siCRBP1-scrambled, (kindly provided by Nicoletta Sacchi, Roswell Park Cancer Institute, NY) were transfected into Phoenix cells using FuGENE transfection reagent (Roche, NSW, Australia). Retroviral supernatants were collected and used to infect HPDE-EcoR cells. Puromycin (1 µg/mL) was used to select successfully infected cells. Successful knockdown of CRBP1 was determined by western blot.

### 3D culture of HPDE cells

HPDE-EcoR cells infected with shRNA were grown on chamber slides coated with growth factor reduced matrigel (BD Biosciences, San Hose, CA) in keratinocyte serum-free medium (Invitrogen, Victoria, Australia) supplemented with epidermal growth factor, bovine pituitary extract, 10% fetal calf serum and 2% matrigel for 20 days. Media was changed every 4 days. 3D structures were recovered at 3, 7, 10, 15 and 20 days using BD cell recovery solution (BD Biosciences, San Hose, CA) and protein extracted for western blot.

### Stable transfection of MiaPaCa2 cells

Four different clones of MiaPaCa2 cells expressing different levels of CRBP1 (MP2^CRBP1^ cells) and their respective controls were created to evaluate the effect of CRBP1 expression on MiaPaCa2 cells. The cells were created using the pcDNA™6.2/GFP plasmid (Invitrogen, Victoria, Australia) and the pQCXIP plasmid (Clontech Laboratories, Mountain, View, CA) containing the CRBP1 gene. The vectors were transfected using Amaxa Nucleofector® Technology (Lonza Cologne AG, Cologne, Germany) according to the manufacturer's instructions. The cells transfected with pcDNA™6.2/GFP plasmid were further sorted for GFP expression using flow cytometry to enrich the population of cells expressing GFP/CRBP1. The expression of CRBP1 in each population was confirmed by western blot. The cells were treated with 2, 5 or 10 mM of all-*trans*-retinoic acid (ATRA; Sigma-Aldrich, St Louis, MA) on Day 1. The cells were harvested and counted on Day 1, 2, 4 and 6 to measure cell proliferation.

### DNA extraction

1 mm tissue cores were removed from formalin-fixed paraffin-embedded human PC samples and DNA was extracted using the Gentra Puregene Tissue Kit (Qiagen, Valencia, CA) according to the manufacturer's instructions. DNA from cell lines was extracted using a DNA extraction kit (Stratagene, Santa Clara, CA) according to the manufacturer's instructions.

### Bisulfite treatment

Bisulfite treatment was carried out using the previously described methods [26] with minor modifications. In summary, 1 µg DNA was denatured by treatment with 0.3 M NaOH and modified with 3 M sodium bisulfite for 6 hours. Modified DNA was purified using QIAEX II Gel Extraction Kit (Qiagen, Valencia, CA). Bisulfite treatment was completed by adding 5.5 ml of 3 M NaOH, precipitated with ethanol, and resuspended in 50 ml of DNA Hydration Solution (Gentra Puregene Tissue Kit, Qiagen, Valencia, CA).

### Methylation specific PCR (MSP)

DNA methylation patterns in the CpG island of CRBP1 in human pancreatic cell lines and human samples were determined by MSP using previously published primers [26], [27]. PCR amplifications were performed in 12.5 µl reaction mixtures containing 1 to 5 µl of bisulfite treated DNA, dNTPs (each at 200 µM), primers (0.4 mM each reaction), 1.5 mM MgCl_2_, 0.75 unit of AmpliTaq Gold® DNA polymerase (Applied Biosystems, Victoria, Australia), and 1× PCR Buffer II (Applied Biosystems, Victoria, Australia). MSP was carried out using the following conditions: 95°C (30 seconds), 58°C (30 seconds), 72°C (30 seconds) for 40 cycles. The cycling conditions included an initial denaturation step at 95°C for 10 minutes and final elongation at 72°C for 10 minutes. All PCR products were visualised using 1.5% agarose gel. MYOD primers [28] were used as a control for the quality of the bisulfite treated DNA. These primers contain no CpG sequences.

### 5-AZA and TSA treatment

MiaPaCa2 cells were treated as follows: (1) Treated on Day 1 with 300 mM of 5-AZA-2′deoxycytidine (5-AZA) (Sigma-Aldrich, St Louis, MO) and harvested on Day 4; (2) Treated on Day 1 with 100 nM of Trichostatin A (TSA) (Sigma-Aldrich, St Louis, MO) and harvested on Day 4; (3) Treated on Days 1 and 3 with TSA and harvested on Day 4; (4) Treated on Day 1 with 5-AZA, Days 2 and 3 with TSA and harvested on Day 4; (5) Treated on Day 1 with 5-AZA, Days 2, 3 and 5 with TSA and harvested on Day 6. Untreated MiaPaCa2 and HPDE cells were used as controls. During treatment, the media was changed daily, and cells were washed twice with cold PBS prior to nucleic acid and/or protein extraction.

## Results

### Retinoid signaling in normal pancreas, pancreatic injury and regeneration

**Figure 1:**
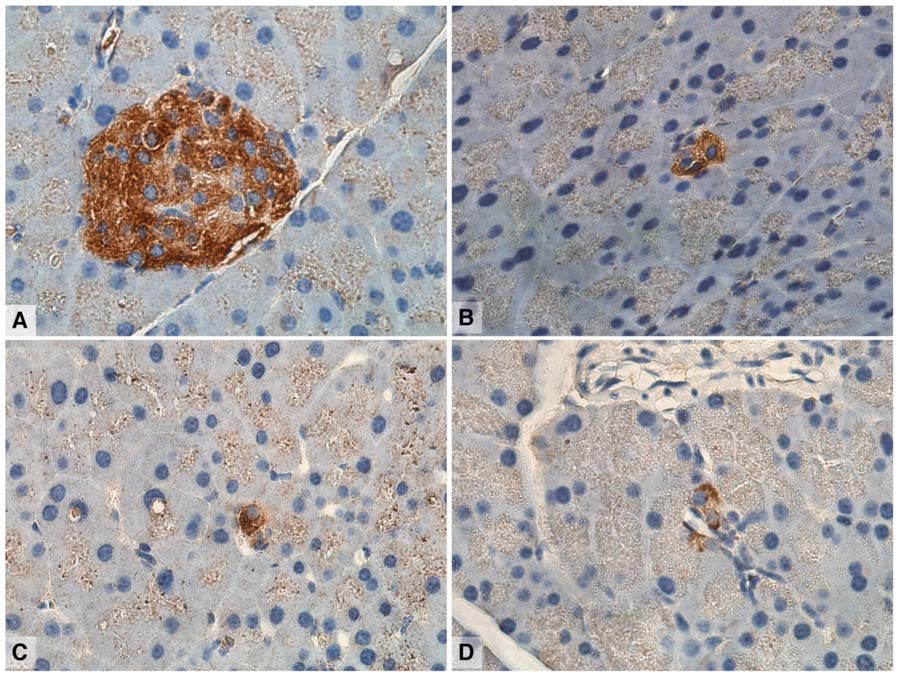
RA signalling activity in adult Retinoic Acid Response Element (RARE-LacZ) reporter mouse pancreas showing positive pancreatic islet (A) and rare positive exocrine cells (B), some of which may represent centro-acinar cells (based on location and morphology) (C–D).

**Figure 2:**
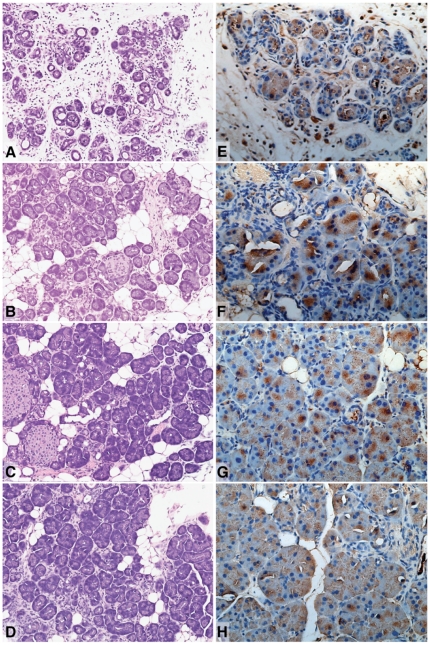
H&E staining of pancreata from RARE-LacZ mice treated with caerulein (A), and subsequent recovery for 2 weeks (B), 4 weeks (C) and 6 weeks (D). RA signalling activity in pancreata from RARE-LacZ mice treated with caerulein (E), and subsequent recovery for 2 weeks (F), 4 weeks (G) and 6 weeks (H). Increased retinoid signaling activity was observed in a significant proportion of acinar cells immediately following cessation of caerulein treatment (E), with retinoid activity peaking at 2 weeks (F), diminishing at 4 weeks (G) and returning to near normal levels following 6 weeks of recovery (H).

Retinoic Acid Response Element (RARE-LacZ) reporter mice that mark cells with active retinoid signaling [29] showed that in normal pancreas, active retinoid signaling is restricted to the Islets of Langerhans and rare single, or small clusters of exocrine cells with an acinar or centro-acinar based on location and morphology (Figure 1). Repeated intraperitoneal injections of the cholecystokinin analogue caerulein [30] induces chronic pancreatitis in mice with morphological changes such as loss of acinar cell mass and an increase in pancreatic fibrosis similar to that seen in the human condition. To investigate retinoid signalling in the setting of pancreatic injury and regeneration, we induced pancreatitis in RARE-LacZ mice. Mice were treated for 10 weeks with intraperitoneal injections of caerulein, sacrificed upon cessation of treatment, or allowed to recover for 2, 4 or 6 weeks. Mice sacrificed immediately following the final injection of caerulein (at the time of acute injury) had significantly smaller pancreata due to acinar cell atrophy and fibrosis with the formation of “tubular complexes”, a manifestation of acinar to ductal metaplasia, the earliest morphological changes associated with pancreatic carcinogenesis (Figure 2A) [31], [32].

At 2 weeks following the cessation of caerulein treatment, exocrine regeneration had commenced with an increase in acinar cell mass, but with residual tubular complexes and a persisting inflammatory infiltrate (Figure 2B). At 4 and 6 weeks, exocrine pancreata had near fully, but not completely regenerated, with occasional acinar units still displaying enlarged lumena and a surrounding mild inflammatory infiltrate (Figures 2C and 2D). β-galactosidase staining of pancreata from caerulein-treated RARE-LacZ mice demonstrated increased retinoid signaling activity in a large proportion of acinar cells immediately following cessation of caerulein treatment (Figure 2E). Retinoid activity peaked at 2 weeks and diminished at 4 weeks with near normal levels at 6 weeks (Figures 2F, G, H).

### Retinoid signalling in pancreatic cancer

**Figure 3:**
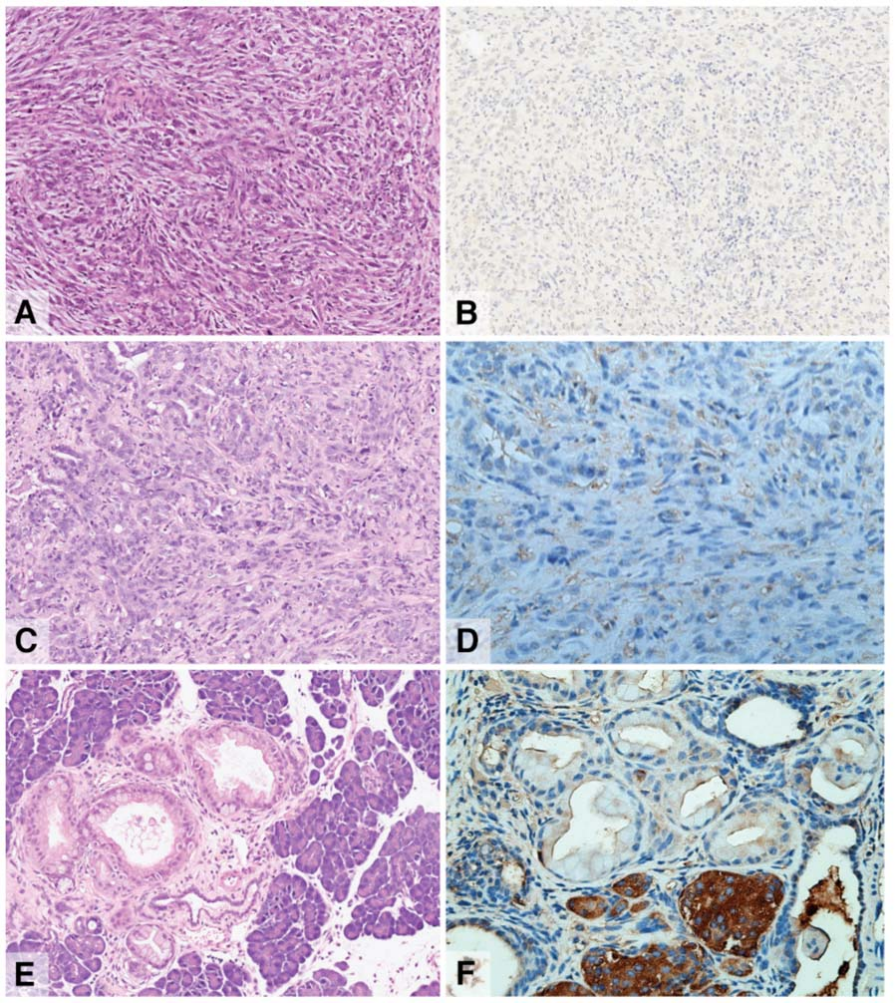
RA signalling activity in DMBA-induced tumours (A–B) and in LSL-KrasG12D/+;LSL-Trp53R172H/+;Pdx1-Cre;RARE-LacZ pancreatic tumours (C–D) and mPanIN lesions (E–F). In tumors from DMBA treated RARE-LacZ mice, a distinct absence of any cells exhibiting active retinoid signaling was observed (B). In LSL-KrasG12D/+;LSL-Trp53R172H/+;Pdx1-Cre;RARE-LacZ pancreatic tumours and precursor lesions, there was also a complete absence of retinoid signaling activity (D and F respectively).

To determine if retinoid signaling in the pancreas was altered during carcinogenesis we investigated retinoid signaling activity in chemically induced (DMBA) [22] and genetically engineered mouse models of pancreatic cancer [33]. DMBA treated RARE-LacZ mice developed poorly differentiated, sarcomatoid tumours (Figure 3A) with a distinct absence of any cells that exhibited active retinoid signalling despite examination of multiple sections (Figure 3B). Pancreas specific promoter driven Cre recombinase (Pdx1-Cre) induced expression of activated KRas and mutant p53 within the pancreas are currently the most appropriate genetically engineered murine model of pancreatic cancer. These LSL-Kras^G12D/+^/LSL-Trp53^R172H/+^/Pdx1-Cre mice develop pancreatic precursor lesions (PanIN) that progress to highly invasive and metastatic pancreatic ductal adenocarcinoma at a median of 5 months [33]. These mice were crossed with RARE-LacZ reporter mice to generate LSL-Kras^G12D/+^/LSL-Trp53^R172H/+^/Pdx1-Cre/RARE-LacZ mice. Pancreatic tumours, which were predominantly moderately-differentiated and contained abundant desmoplastic stroma formed in all mice. There was again a complete absence of retinoid signalling activity in these tumours and in precursor lesions despite multiple sectioning and assessment by 2 independent observers, one of whom was a specialist pancreatic pathologist (Figure 3D, E,F).

### Loss of CRBP1 expression is a common and early event in pancreatic cancer

Cellular Retinoid Binding Protein 1 (CRBP1), a key regulator of retinoid signaling is thought to play a significant role in breast carcinogenesis [16], [34]. Preliminary observations also identified downregulation of CRBP1 transcript levels in PC [35] and PanIN [36]. As a consequence, we investigated loss of CRBP1 as a potential cause of diminished retinoid signaling in PC, and a role in pancreatic carcinogenesis.

**Figure 4:**
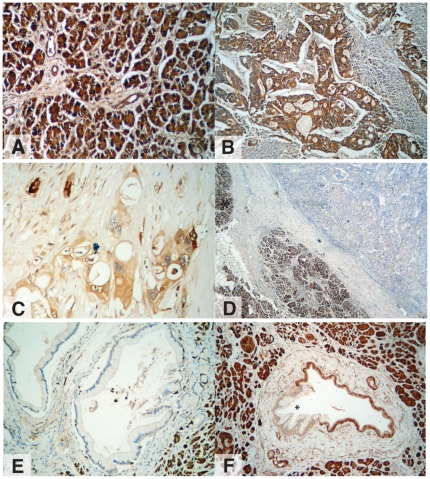
CRBP1 expression in (A) normal pancreas, (B) PC positive for CRBP1, (C) PC demonstrating partial loss of CRBP1 expression, (D) PC completely lacking CRBP1 expression, (E) PanIN-1A lesion negative for CRBP1 expression, (F) PanIn-1B (*) lesion negative for CRBP1 expression with positive adjacent normal ductal epithelium.

**Figure 5:**
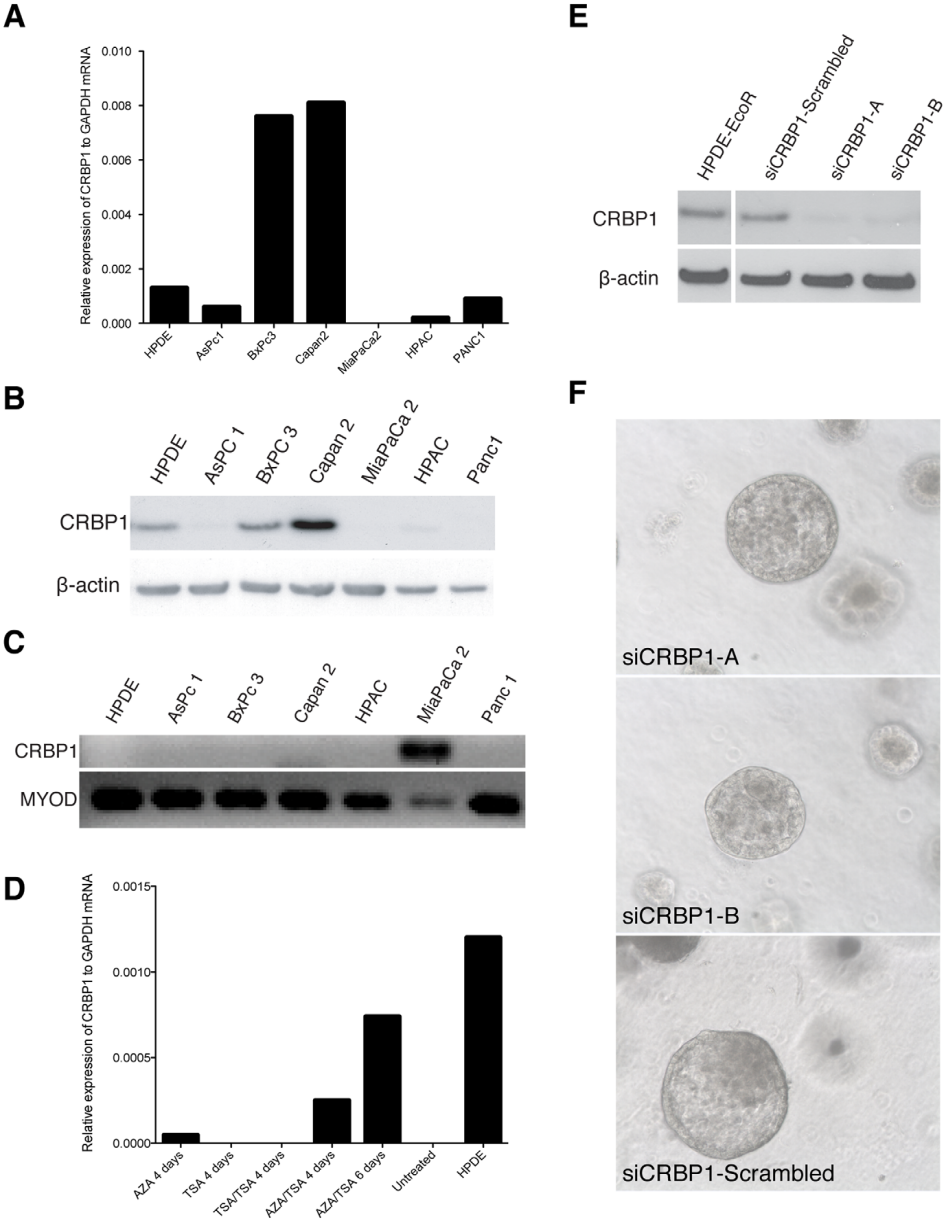
mRNA expression (A) and protein expression (B) of CRBP1 in PC cell lines. (C) CRBP1 methylation status in PC cell lines and (D) restoration of CRBP1 expression with combination treatment of MiaPaCa2 cells with 5-Aza and TSA. (E) CRBP1 knockdown in stably transfected HPDE-EcoR cells. (F) HPDE cells grown in 3D demonstrate no change in morphology between siCRBP1 cells and scrambled control.

In a cohort of 90 patients, immunohistochemistry showed significant downregulation of CRBP1 protein expression in 70%, with complete loss of expression in 50% of these (35% of total; Figure 4A, B, C,D). Loss, or downregulation of CRBP1 expression occurred early in PC development and was present in 100% of PanIN-1A and 1B lesions of patients who had aberrant expression within their cancer (Figure 4E, F). Loss of CRBP1 expression also occurred in 50% of early precursor lesions (PanIN1A and 1B) associated with chronic pancreatitis, a known risk factor for PC. Neither loss, nor downregulation of expression was associated with patient outcome (Log-Rank *P* = 0.3539; Figure S1). In addition, 4 of the 6 PC cell lines demonstrated loss or downregulation of CRBP1 expression (Figure 5A and B).

### Loss of CRBP1 expression is associated with reversible promoter hypermethylation

Methylation specific PCR (MSP) and bisulfite genomic sequencing (BSG) identified CRBP1 promoter hypermethylation in 27.3% of 33 human PC samples lacking CRBP1 expression in contrast to 5 of 5 matched normal pancreas samples which were not methylated. CRBP1 promoter hypermethylation was detected in MiaPaCa2 cells (Figure 5C), and treatment with the demethylating agent 5-AZA alone, or in combination with the HDAC inhibitor TSA, induced expression of CRBP1 mRNA (Figure 5D). The ability to reverse CRBP1 silencing pharmacologically presented the potential opportunity to use retinoids and HDAC inhibitors as combination therapies to therapeutically target CRBP1.

### Downregulation of CRBP1 expression in cell lines and mouse models

To assess the role of CRBP1 downregulation in pancreatic carcinogenesis, immortalized Human Pancreatic Ductal Epithelial cells expressing the mouse ecotropic retroviral receptor (HPDE-EcoR) were retrovirally transduced with two shRNA constructs targeting CRBP1 as well as a scrambled control. Cells were then grown in 3D culture for 20 days. Western blot demonstrated 50% knockdown of CRBP1 expression (Figure 5E), however, there was no discernible difference in morphology compared to controls (Figure 5F). In addition, pancreata from 12 month-old CRBP1 knockout mice [37] (kindly provided by Prof. Pierre Chambon) revealed no obvious morphological differences compared to control mice (data not shown).

### CRBP1 overexpression in MiaPaCa2 cells

In order to examine the effects of restoration of CRBP1 expression, MiaPaCa2 cells, which normally do not express CRBP1 were stably transfected to generate several clones expressing different levels of CRBP1 (low, medium, high and very high) with no effect on the proliferation rates compared to the empty vector control (Figure S2A) and did not alter sensitivity to retinoid therapy when treated with All-trans Retinoic Acid (AtRA) (Figures S2B and S2C).

## Discussion

The emerging role of dysregulated embryonic signaling pathways such as Notch and Hedgehog [4] is providing opportunities for the development of novel therapeutic strategies for pancreatic cancer. Retinoid signaling is indispensible for normal embryonic development, including the formation of the nascent pancreas [6], [7], [8]. Our data show that signaling activity in normal pancreas is restricted to pancreatic islets and rare cells in the exocrine pancreas, many of which resembled centro-acinar cells, the putative stem cells of the pancreas. A recent study identified that cells in the pancreas expressing the RA-synthesising enzyme, aldehyde dehydrogenase 1 (ALDH1), are centroacinar cells that exhibit progenitor cell characteristics [38]. This pattern of activity is similar to the expression of the transcription factor Pdx1 which is also thought to mark exocrine progenitor or “stem” cells. Functional readouts of *in vivo* retinoid signaling activity using reporter mice in our study suggest that augmented signaling plays a role in pancreatic regeneration after injury. Active signaling, normally restricted to specific cell types becomes widespread until differentiation of new exocrine acini is complete. Treatment of mice with repeated doses of caerulein results in a dramatic increase in ALDH1-positive cells, which is assumed to represent increased retinoid signaling [38], further supporting our findings.

Deregulated retinoid signaling has been identified in many cancer types [11], and although previous studies have identified aberrant expression of components of retinoid signaling as well as downstream targets in PC [35], [39], [40], [41], [42], [43], little was known concerning retinoid signaling activity. Assessment of retinoid signaling reporter activity in both chemically induced pancreatic cancer, and in genetically engineered models in our study demonstrated a lack of retinoid signaling. Based on these observations, we hypothesized that a lack of retinoic acid signaling may impede normal differentiation in response to carcinogenic stimuli and contribute to pancreatic carcinogenesis, and that restoration of retinoid signaling may provide a novel therapeutic option.

As a consequence, we investigated the role of CRBP1, a key component of retinoid signaling, and thought to play a major role in breast carcinogenesis [16], [34]. We identified loss, or downregulation of CRBP1 expression (which would potentially confer loss of retinoid signaling activity) in 70% of pancreatic cancer specimens, with a high proportion due to promoter methylation. Loss or downregulation of CRBP1 expression was present in the earliest precursor lesions of PC, both in association with PC and in chronic pancreatitis, a risk factor for the development of PC. However, knockdown of CRBP1 expression in immortalised pancreatic ductal epithelial cells did not induce transformation. Similarly, CRBP1 knockout mice did not demonstrate a pancreatic phenotype at over 12 months of age. In addition, reintroduction of CRBP1 in PC cells deficient in CRBP1 did not alter insensitivity to retinoid therapy. Importantly, recent data suggest that pancreatic stellate cells may play a key role in retinoid signaling and resistance to retinoid therapy *in vivo* [44]. This needs to be taken into consideration, particularly as the data presented here focused on the pancreatic epithelial component.

In summary, pancreatic injury induces retinoid signaling activity within the pancreas, which is widespread during injury and regeneration and potentially plays an important role in this process. The lack of retinoid signaling activity in mouse models of pancreatic cancer suggests an important role in pancreatic carcinogenesis, however, despite the high prevalence of loss of CRBP1 expression, and its key role in other cancers, there is no evidence to support that loss of CRBP1 is alone sufficient to alter retinoid signaling, or induce carcinogenesis in the model systems used in this study.

## Supporting Information

Figure S1 **Kaplan-Meier survival curve for CRBP1 expression in PC.**
(TIF)

Figure S2 **Cell proliferation assay of MiaPaCa2 cells transfected with different levels of CRBP1 (A).** Effect of retinoid treatment on (B) MiaPaCa2 cells; (C) MiaPaCa2 cells transfected with CRBP1; and (D) HPDE cells. MiaPaCa2 cells were resistant to AtRA treatment, despite the re-introduction of CRBP1, while HPDE cells were sensitive to AtRA treatment.
(TIF)
