# Supplementary figures and images for: Retinoid Signaling in Pancreatic Cancer, Injury and Regeneration
Source: PLoS One. 2011 Dec 29;6(12):e29075. doi: 10.1371/journal.pone.0029075 (PMC3248409; doi:10.1371/journal.pone.0029075)

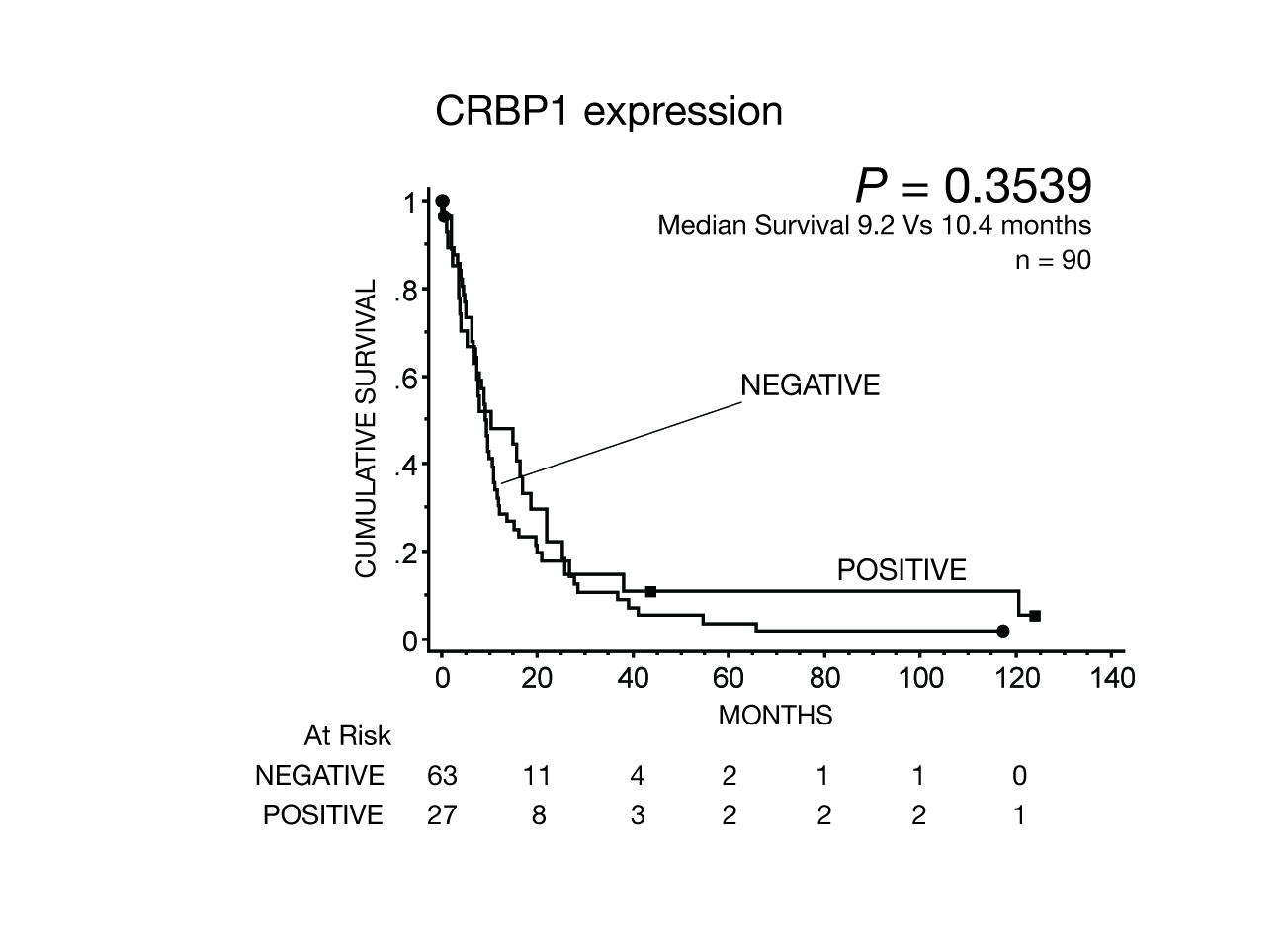

Supplement: Figure S1 — Kaplan-Meier survival curve for CRBP1 expression in PC. (TIF) [file pone.0029075.s001.tif]

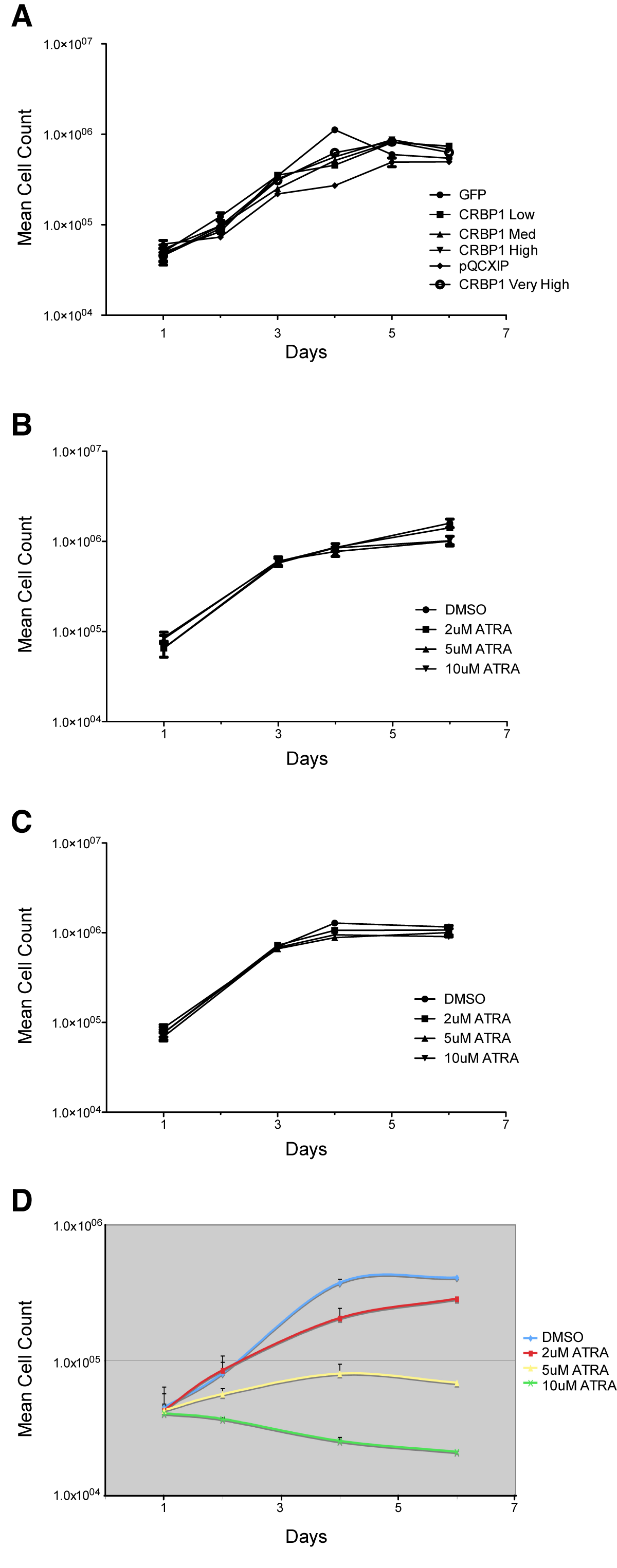

Supplement: Figure S2 — Cell proliferation assay of MiaPaCa2 cells transfected with different levels of CRBP1 (A). Effect of retinoid treatment on (B) MiaPaCa2 cells; (C) MiaPaCa2 cells transfected with CRBP1; and (D) HPDE cells. MiaPaCa2 cells were resistant to AtRA treatment, despite the re-introduction of CRBP1, while HPDE cells were sensitive to AtRA treatment. (TIF) [file pone.0029075.s002.tif]
